# A novel polyp retrieval bag reduces the polyp fragmentation rate in colon polypectomy: a single-blind randomized controlled study

**DOI:** 10.1007/s00384-024-04694-9

**Published:** 2024-07-24

**Authors:** Jindong Chu, Cuiyun Ma, Min Min, Qian Bi, Wei Shen, Xueting Zhang, Hanqing Zhang, Aitong Li, Yan Liu, Zheng Lu

**Affiliations:** 1 Senior Department of Hepatology, the Fifth Medical Center of PLA General Hospital, Beijing, 100039 China; 2 Senior Department of Gastroenterology, the First Medical Center of PLA General Hospital, Beijing, 100071 China

**Keywords:** Colonic polyps, Colonoscopy, Polyp retrieval, Fragmentation

## Abstract

**Purpose:**

The fragmentation of polyps affects complete resection confirmation. The primary aim of this study was to assess the feasibility of a novel polyp retrieval bag for reducing the fragmentation rate of colon polyps.

**Methods:**

Patients with a 5–15 mm colon polyp were recruited and randomized into two groups at a 1:1 ratio. After polyp resection, polyps obtained from patients in the treatment group were extracted via a novel polyp retrieval bag without traversing the instrument channel, whereas polyps obtained from patients in the control group were collected through the instrument channel, attaching the polyp trap to the instrument channel port, and applying suction.

**Results:**

From January to July 2022, 225 patients were assessed for eligibility. The study participants included 204 patients, and seven patients whose samples were not retrieved were excluded. Polyp fragmentation was significantly lower in the treatment group than in the control group (3.0% [3/100] vs. 17.5% [17/97], *P* = 0.001). The retrieval failure rates in the treatment group and control group were not significantly different (2.0% [2/102] vs. 4.9% [5/102], *P* = 0.442). There were fewer colonoscope insertions in the treatment group than in the control group (102 vs. 110), but a significant difference was not present (*P* = 0.065). No significant adverse events were observed during the follow-up.

**Conclusions:**

This study demonstrated that the polyp retrieval bag was safe and feasible for reducing the fragmentation rate of retrieved polyps.

**Trial registration:**

The study was registered at ClinicalTrials.gov (NCT05189912, 1/12/2021).

## Introduction

The resected polyp must be retrieved for histopathological diagnosis and complete resection confirmation after polyp resection [1]. Depending on the size of the polyps, they may be retrieved either by aspiration through the channel of a colonoscope or by the use of an endoscopic retrieval device. In most cases, small polyps that are 15 mm or less in diameter can be retrieved through the channel of the colonoscope via different methods, including the conventional method of pressing the suction valve button and retrieving the polyp through a polyp trap affixed to the suction connector at the light guide connector section of the colonoscope; similar to the conventional method, but removing the suction valve and sealing the open suction valve cylinder with a finger; using a Roth Net polyp retriever placed and then removed through the instrument channel; or connecting the polyp trap to the instrument channel port [2]. Large polyps that are > 15 mm can be recaptured by polyp retriever nets or other devices, such as small grip-seal plastic bags [3] and polypectomy snares [4], and can be recovered by withdrawal of the colonoscope.

However, previous reports have indicated that many polyps retrieved while passing through the channel of a colonoscope are fragmented, leading to a polyp fragmentation rate of 18.5 ~ 60.3% [2, 5]. This serious issue makes it difficult for pathologists to accurately determine the completeness of resection and submucosal invasion [6].

To retrieve resected polyps more efficiently and reduce polyp fragmentation, we designed a self-expanding polyp retrieval bag. The polyp retrieval bag has the ability to retrieve one or more polyps, and they do not traverse the biopsy channel after polyp retrieval, thereby reducing the rate of polyp fragmentation. The method chosen for control in this study was the one described in the literature with the lowest rate of polyp fragmentation while traversing instrument channels. This method involves connecting the polyp trap to the instrument channel port with suction applied [2]. In this study, fragmentation of polyps extracted via the above two methods was assessed.

## Methods

### Patients

Patients who underwent colonic polypectomy were included in this single-center randomized controlled trial. We included adults aged at least 18 years with a colonic polyp 5–15 mm in size. If the patient presented with multiple eligible polyps, only the first eligible polyp that was detected was chosen for inclusion. The exclusion criteria included intolerance to endoscopy; significant cardiopulmonary, hepatic, or renal conditions (such as acute myocardial infarction, heart failure, respiratory dysfunction, liver function classified as Child‒Pugh B or C, liver or renal failure); underlying bleeding disorders; non-en bloc removal of polyps; a platelet count lower than 50 × 10^9^/L; pregnancy; and other high-risk conditions or diseases (e.g., massive ascites, uncontrolled infection, serious electrical disturbances, or hemoglobin levels below 60 g/L). The exclusions were implemented as part of our standard practice and were not limited to this particular study. It conforms to the Consolidated Standards of Reporting Trials (CONSORT). In accordance with the Declaration of Helsinki, all patients enrolled in the study provided written informed consent after approval by the institutional review board of the Fifth Medical Center of PLA General Hospital. The study was registered at ClinicalTrials.gov (NCT05189912, 1/12/2021). Patient demographics and clinical data, including age, sex, quality of bowel preparation, pathological diagnosis, polyp size, location, morphology, resection methods, and retrieval failures, were collected for all patients.

### Randomization and masking

Computer-generated random numbers were used to divide eligible patients into two groups at a 1:1 ratio. A numbered opaque sealed envelope was used for randomization. Patients were blinded to the randomization. Two endoscopists performed polyp resection and retrieval. The polyps were resected via a cold snare or endoscopic submucosal resection (EMR) according to the endoscopists’ experience. The specimens were reviewed independently by two experienced pathologists who were not informed about the patient randomization. A 14-day follow-up was conducted after polyp resection to observe for any adverse events.

### Methods of polyp retrieval

**Fig. 1:**
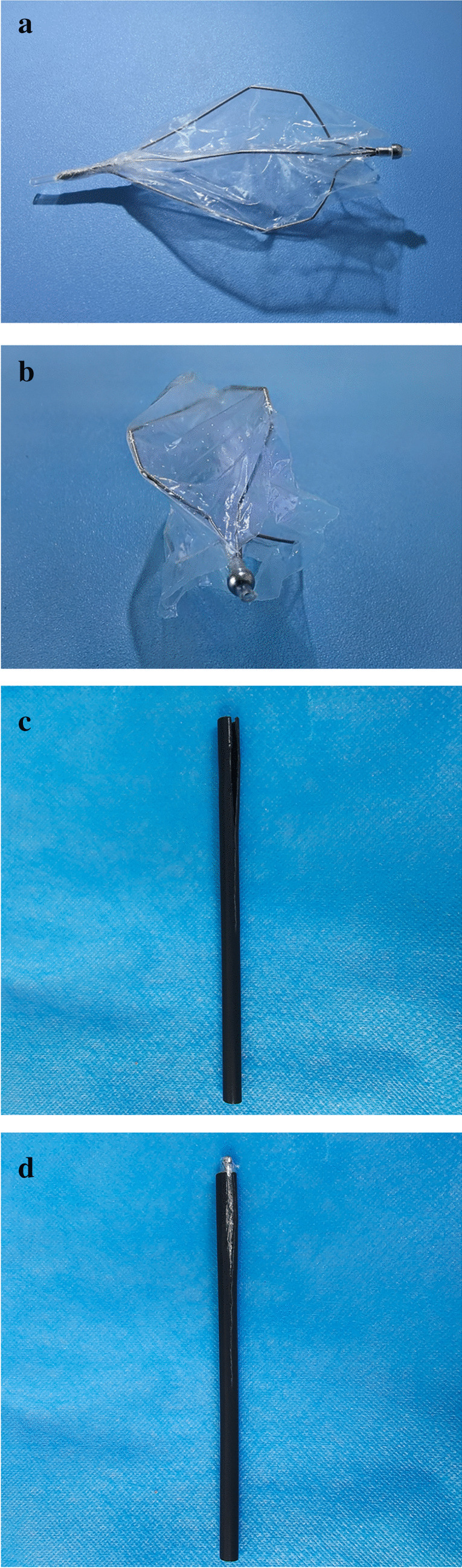
The polyp retrieval bag. **a** Top view of the bag. **b** Frontal view of the bag. **c** The partially split plastic cannula. **d** The bag enclosed in the plastic cannula

**Fig. 2:**
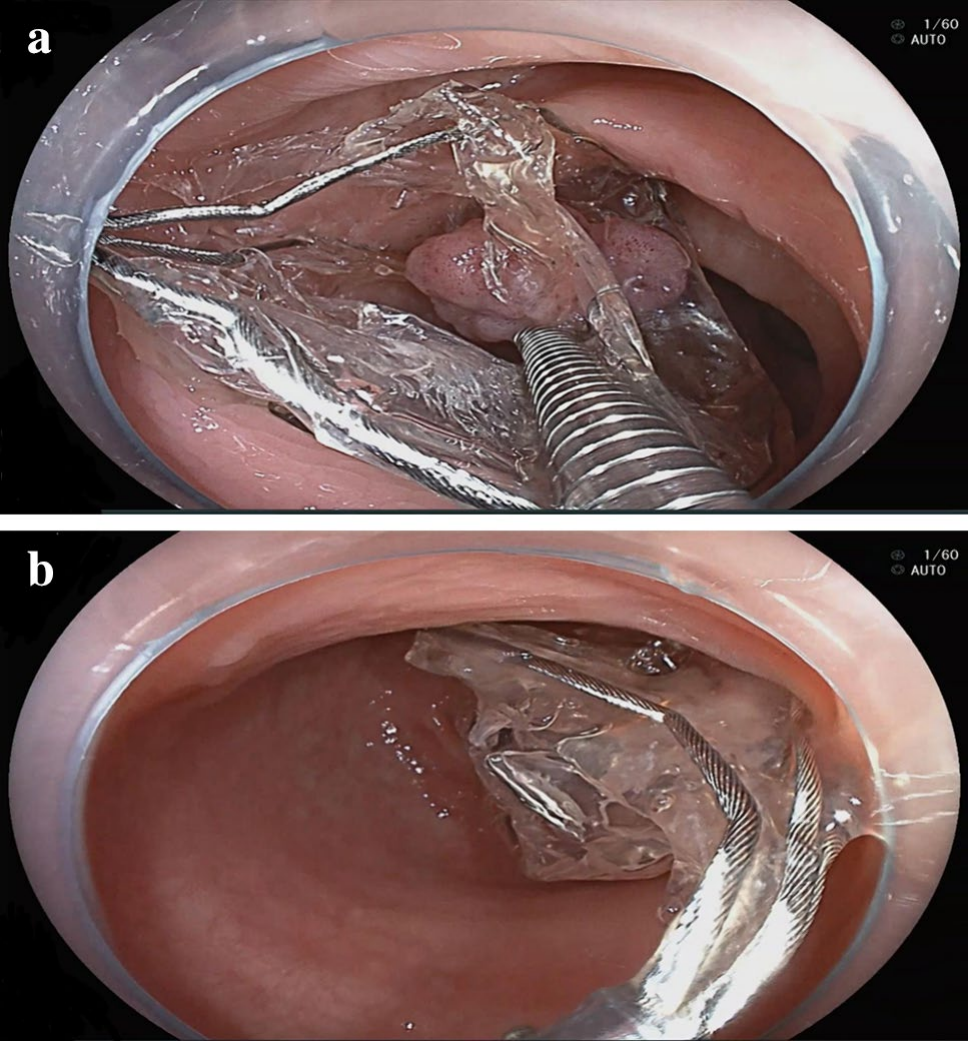
A resected polyp retrieved by the polyp retrieval bag. **a** A resected polyp was placed into the polyp retrieval bag with a hemostatic clip. **b** The metal top of the polyp retrieval bag was grasped by a snare and pulled out with a colonoscope

The polyps in the two groups were retrieved via two methods. In the experimental group, a polyp retrieval bag was employed, which was constructed via a self-expanding stone extraction basket (Micro-Tech, Nanjing, China) covered with a plastic film, measuring 5.5 cm × 3.0 cm in length and width, as illustrated in Fig. 1a and b. The bag had a narrow opening but a spacious inner compartment. Typically, a plastic film matching the dimensions of the expanded stone extraction basket was securely attached to the basket’s framework via a heating rod. The plastic film covered all metallic components of the basket except for the top section. To aid in the insertion of the polyp retrieval bag into the instrumental channel, a partially split plastic cannula of approximately 5 cm was used to enclose the bag, as depicted in Fig. 1c and d. Following polyp resection, the cannula-and-bag assembly was introduced into the opening of the instrumental channel and advanced through the channel via biopsy forceps. Subsequently, the bag automatically unfolded after being placed in the colon. The resected polyps were then placed into the bag by using a hemostatic clip (Micro-Tech, Nanjing, China) or biopsy forceps (Lookmed, Changzhou, China) (Fig. 2a). The selection of either a biopsy forceps or a hemostatic clip for clipping resected polyps to the bag was based on the size or texture of the polyps. Biopsy forceps were chosen for small or hard-textured polyps, whereas a hemostatic clip was preferred for larger or fragile-textured polyps to prevent polyp fragmentation during retrieval procedures. After the procedure was complete, the metal top of the polyp retrieval bag was grasped by a polypectomy snare (MTW-Endoscopie, Sebastian, Germany) and pulled close to the colonoscope. The opening of the bag was shrunken (Fig. 2b) before it was pulled out of the colon with a colonoscope (Fujifilm Co., Tokyo, Japan). The control group involved connecting a polyp trap (e-LinkCare Meditech Co., Hangzhou, China) onto the instrument channel port with suction applied [2]. The rationale for choosing the method in the control group is that it has the lowest fragmentation rate among the four methods in previous studies [2].

### Assessment of polyp fragmentation

According to Barge W et al. [2] and Kishida Y et al. [5], the presence of multiple pieces in the gross description of a specimen in formalin is defined as polyp fragmentation. The polyps were reviewed for fragmentation less than 2 h after removal from patients.

### Primary and secondary outcomes

In this study, the polyp fragmentation rate was the primary outcome. Polyp retrieval failure rates, colonoscope insertions, and adverse events, including delayed bleeding, delayed perforation, and postpolypectomy syndrome, were the secondary outcomes. Polyp retrieval failure is defined as failure to retrieve a polyp by the allocated retrieval method or the absence of resected polyps. If a polyp was missed or could not be retrieved via the allocation method, it was excluded from the analysis, retrieved via an alternative technique, and withdrawn via the colonoscope or aspiration through the suction channel of the colonoscope.

### Statistical analysis

On the basis of the primary outcome measure of a decreased polyp fragmentation rate, the sample size calculation utilized the *t* test for two independent samples. According to the design principles, the polyp fragmentation rate of the treatment group was lower than 1%, whereas the lowest polyp fragmentation rate for the control group was reported to be 18.5% [2]. The study needed 86 patients in total (43 patients in each group) to detect a difference with 80% power (assuming a two-sided significance level of 5%). On the basis of previous reports [7], the recruitment target was increased to 102 patients (51 patients in each group) to account for retrieval failure rates of 10–15%.

The researchers voiced concerns about a relatively low fragmentation rate in the control group, and an interim analysis of the study was conducted after 74 patients had been recruited. A new target sample size was increased to 196 patients to reduce the fragmentation rate from 12.0% (the control group) to 2.5% (the treatment group). The study was stopped once the sample size reached 204 patients. SAS 9.4 was used to calculate the sample size.

The categorical variables are presented as frequencies and percentages and were compared via Fisher’s exact tests, χ2 tests, or continuously corrected χ2 tests. The means and standard deviations (SDs) are presented for continuous variables, and independent *t* tests were used for comparisons. Statistical analyses were conducted via SPSS version 26 with two-sided *P* values. A *P* value of < 0.05 was considered significant in all the cases. To analyze risk factors, logistic regression analysis was applied for multivariate regression. The statistical analysis was performed via R statistics version 3.5.3.

## Results

### Patients

**Fig. 3:**
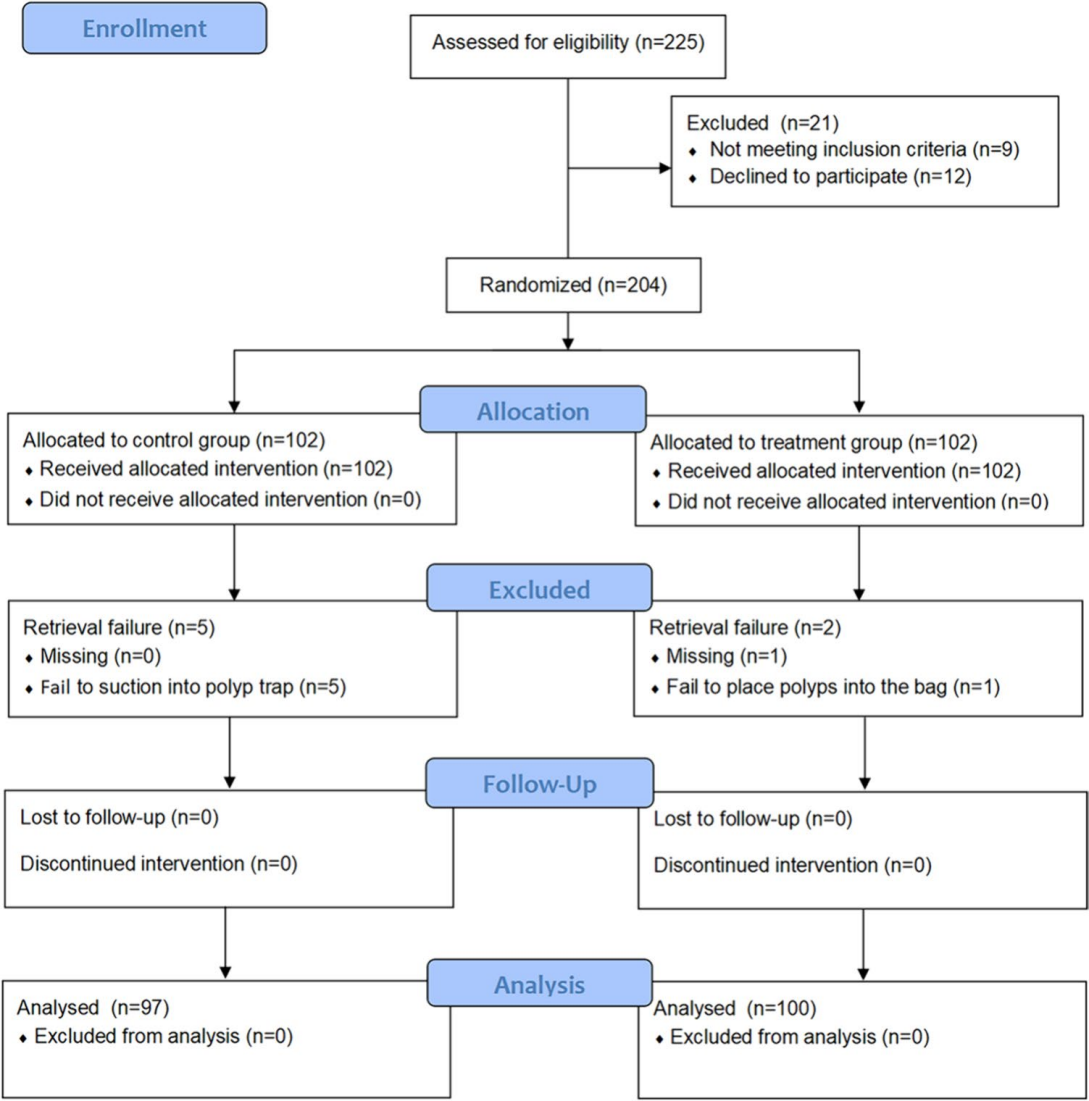
Flow chart of patient enrollment

A total of 225 patients were assessed for study eligibility from January 17, 2022, to July 30, 2022. The study participants included 204 patients, seven of whom were excluded because of failed specimen retrieval. Ultimately, 97 patients in the control group and 100 patients in the treatment group were included in the final analysis (Fig. 3). Patients and polyps were compared on the basis of their baseline characteristics, as shown in Table 1. In both groups, the patient characteristics and polyp features were similar.

**Table 1:** Characteristics of patients and polyps

	Control group	Treatment group	*P* value
	Polyp trap attached to instrument channel port *n* = 97	Polyp retrieved bag *n* = 100	
Sex, *n* (%)			0.769
Male	68 (70.1)	72 (72.0)	
Female	29 (29.9)	28 (28.0)	
Age, y, mean (SD)	60.2 (10.2)	60.4 (10.9)	0.919
Polyp location, *n* (%)			0.076
Proximal*	46 (47.4)	35 (35.0)	
Distal	51 (53.6)	65 (65.0)	
Size, *n* (%)			0.630
5–9 mm	46 (47.4)	44 (44.0)	
10–15 mm	51 (52.6)	56 (56.0)	
Morphology, *n* (%)			0.897
Flat	22 (22.7)	26 (26.0)	
Sessile	67 (69.1)	66 (66.0)	
Pedunculated	8 (8.2)	8 (8.0)	
Pathology, *n* (%)			0.195
Tubular adenoma	65 (67.0)	78 (78.0)	
Tubulovillous adenoma	3 (3.1)	4 (4.0)	
Hyperplastic	28 (28.9)	17 (17.0)	
Serrated adenoma	1 (1.0)	1 (1.0)	
Quality of bowel preparation, *n* (%)			0.780
Excellent	65 (67.0)	72 (72.0)	
Good	23 (23.7)	20 (20.0)	
Fair	9 (9.3)	8 (8.0)	
Resection method, *n* (%)			0.500
EMR	73 (75.3)	71 (71.0)	
Cold snare	24 (24.7)	29 (29.0)	
Endoscopist, *n* (%)			0.518
A	52 (53.6)	49 (49.0)	
B	45 (46.4)	51 (51.0)	
Fragmentation rates, *n* (%)	17 (17.5)	3 (3.0)	0.001
Retrieving failure rates, %	4.9 (5/102)	2.0 (2/102)	0.442
Colonoscope insertions, *n*	110	102	0.065
1 insertion	95	101	
2 insertions	6	1	
3 insertions	1	0	
Adverse events	0	0	-

*SD *standard deviation, *EMR *endoscopic submucosal resection

*Including cecum, ascending colon, and hepatic flexure

### Primary outcome

The fragmentation rate of the treatment group was significantly lower than that of the control group (3.0% [3/100] vs. 17.5% [17/97], *P* = 0.001) (Table 1). Endoscopists, polyp location, polyp size, polyp morphology, polyp pathology, or resection methods did not significantly influence the fragmentation rates (Table 2). When the fragmentation rates in the two groups were analyzed on the basis of differences in bowel preparation quality, there was no significant difference in fragmentation rates within the control group (18.5% [12/65, excellent] vs. 13.0% [3/23, good] vs. 22.2% [2/9, fair], *P* = 0.699). Nevertheless, bowel preparation quality had a significant effect on fragmentation rates, as the rates of fragmentation were 1.4% (1/72, excellent), 0% (0/20, good), and 25.0% (2/8, fair) (*P* = 0.033). However, considering that only three polyps were fragmented in the treatment group, the applicability of these findings was limited. The interobserver concordance of polyp macroscopic fragmentation between the two pathologists was 100%.

**Table 2:** Fragmentation rates of two poly retrieval methods

	Fragmentation rate (%)			
	Control group	*P* value	Treatment group	*P* value
	Polyp trap attached to instrument channel port		Polyp retrieved bag	
Total polyps	17.5 (17/97)		3.0 (3/100)	
Polyp location		0.270		> 0.999
Proximal*	13.0 (6/46)		2.9 (1/35)	
Distal	21.6 (11/51)		3.1 (2/65)	
Size		0.570		> 0.999
5–9 mm	15.2 (7/46)		0 (0/44)	
10–15 mm	19.6 (10/51)		5.4 (3/56)	
Morphology		0.426		0.655
Flat	22.7 (5/22)		0 (0/26)	
Sessile	17.9 (12/67)		4.5 (3/63)	
Pedunculated	0 (0/8)		0 (0/8)	
Pathology		0.647		> 0.999
Tubular adenoma	18.5 (12/66)		3.8 (3/78)	
Tubulovillous adenoma	33.3 (1/3)		0 (0/4)	
Hyperplastic	14.3 (4/28)		0 (0/17)	
Serrated adenoma	0 (0/1)		0 (0/1)	
Quality of bowel preparation		0.699		0.033
Excellent	18.5 (12/65)		1.4 (1/72)	
Good	13.0 (3/23)		0 (0/20)	
Fair	22.2 (2/9)		25.0 (2/8)	
Resection method		0.455		0.554
EMR	19.2 (14/73)		4.2 (3/71)	
Cold snare	12.5 (3/24)		0 (0/29)	
Endoscopist		0.551		0.972
A	15.4 (8/52)		4.1 (2/49)	
B	20.0 (9/45)		2.0 (1/51)	

*EMR *endoscopic submucosal resection

*Including cecum, ascending colon, and hepatic flexure

Logistic analysis of the fragmentation rate in the control group did not reveal risk factors (including sex, age, polyp location, quality of bowel preparation, polyp size, morphology, pathology, resection method, and different endoscopists) that could have affected the outcome of fragmentation (Table 3).

**Table 3:** Relationship of each factor with polyp fragmentation in the control group (univariate analysis)

Characteristics		Polyps fragment/intact (*n*) (17/80)	OR (95% CI)	*P* value
Sex	Male	11/57	Reference	
	Female	6/23	1.35 (0.45–4.09)	0.593
Age, y, mean(SD)		61.2 (7.7)/60.1 (10.6)	1.01 (0.96–1.07)	0.677
Polyp location	Proximal*	6/40	Reference	
	Distal	11/40	1.36 (0.47–3.93)	0.571
Quality of bowel preparation	Excellent	12/53	0.79 (0.15–4.30)	0.788
	Adequate	3/20	0.53 (0.07–3.82)	0.525
	Inadequate	2/7	Reference	
Size	5-9 mm	7/39	Reference	
	10-15 mm	10/41	1.36 (0.47–3.93)	0.571
Morphology	Flat	5/17	Reference	
	Sessile	12/55	0.74 (0.23–2.41)	0.619
	Pedunculated	0/8	< 0.01 (0–infinite)	0.991
Pathology	Tubular adenoma	12/54	Reference	
	Tubulovillous adenoma	1/2	2.21 (0.18–26.39)	0.531
	Hyperplastic	4/24	0.74 (0.22–2.52)	0.625
	Serrated adenoma	0/1	< 0.01 (0–infinite)	0.992
Resection method	EMR	14/59	1.66 (0.43–6.36)	0.459
	Cold snare	3/21	Reference	
Endoscopist	A	8/44	Reference	
	B	9/36	1.38 (0.48–3.93)	0.552

*EMR* endoscopic submucosal resection

*Including cecum, ascending colon, and hepatic flexure

### Secondary outcomes

Five polyps were hard to retrieve by suctioning them into the polyp trap through the instrument channel port, which were deemed retrieval failures, but they were captured within a polypectomy snare and recovered by withdrawal of the colonoscope. One polyp 5 mm in size was difficult to drop into the polyp retrieval bag and was recovered via biopsy forceps. One polyp 5 mm in size that was allocated to the treatment group was missed before being placed into the polyp retrieval bag. There was no significant difference in the retrieval failure rates between the treatment group and the control group (2.0% [2/102] vs. 4.9% [5/102], *P* = 0.442) (Table 1). Although there were fewer colonoscope insertions in the treatment group than in the control group (102 vs. 110), a significant difference was not present (*P* = 0.065) (Table 1). No significant adverse events, such as postpolypectomy syndrome, delayed bleeding or delayed perforation, were observed during the follow-up in either group.

## Discussion

Because of the risk of developing colorectal cancer, colonoscopic removal of precancerous polyps is recommended [8]. However, incompletely resected polyps are an important clinical issue and have been implicated in the development of interval colorectal cancer [9, 10], which has a significantly increased risk of death [11]. The completeness of resected polyps is crucial for pathologists. Conventional and advanced methods for retrieving polyps involve traversing the instrumental channel [2, 5], leading to potential polyp fragmentation. A new method, the water-slider method, surrounds polyps with water from an auxiliary water channel during suctioning, with a reported polyp fragmentation rate of 8.2% for smaller polyps (≤ 10 mm) [12]. Other methods utilize additional devices, such as grip-seal plastic bags [3] and the Roth retrieval net [4]. However, there are no available data on the polyp fragmentation rate for grip-sealed plastic bags and retrieval nets.

We hypothesized that the retrieved polyps would not be fragmented if they were not retrieved through a colonoscope channel. We invented and applied the polyp retrieval bag in polypectomy; this was modeled after the success of the specimen retrieval bag in laparoscopic appendectomy [13, 14]. Unlike other methods, the polyp retrieval bag cannot pass through the colonoscope channel after polyp retrieva. The polyp retrieval bag is designed with a large volume, small opening, and insulation, which allows easy manipulation. The plastic film is used for insulation to avoid electrical injury, the self-expanding metal basket is used to avoid folding of the plastic film and is convenient for delivery in the instrument channel, and the fusiform designation ensures a large volume and small opening. Moreover, since the long axis of the expanding polyp retrieval bag is parallel to the colonic lumen, it is easy to place resected polyps into the bag with hemostatic clips or biopsy forceps. Therefore, manipulation of the polyp retrieval bag is straightforward for endoscopists. In contrast to the retrieval net, the polyp retrieval bag does not occupy the instrumental channel. Owing to these advantages, the polyp retrieval bag is considered appropriate for the retrieval of large polyps, multiple polyps, and specimens obtained from piecemeal endoscopic mucosal resection. This helps avoid the need for repeated colonoscope insertions.

This size of polyp was chosen in this study because it can easily be suctioned through the colonoscopy instrument channel, which is related to the control method. Through the colonoscopy instrument channel, polyps that are smaller than 5 mm can easily be removed and retrieved with biopsy forceps, whereas polyps that are larger than 15 mm are difficult to remove with suction. The polyp fragmentation rate in the treatment group was significantly lower than that in the control group in this study. Nevertheless, three polyps in the treatment group were fragmented after retrieval. At least two potential explanations exist to explain this. First, the use of forceps or clips to secure polyps results in their destruction. Second, the anal sphincter crushes polyps in the bag. In addition, one polyp retrieval failure in the treatment group was recorded in this study, and it was more difficult to retrieve small polyps. The elimination of polyps after opening the forceps relies on gravity; therefore, it was easy to drop large polyps. However, small polyps are prone to adhering to biopsy forceps, which makes it difficult to drop them into the bag. This is one of the limitations of the polyp retrieval bag. Another limitation is that the opening size of the bag is limited, and polyps with a hard texture or polyps that are ≥ 2 cm may not be suitable for retrieval. Therefore, the polyp retrieval bag is suitable for retrieving polyps 5–20 mm in size. Moreover, the manipulation of the polyp retrieval bag is more complex than the conventional method is.

We also acknowledge several limitations in our study. One limitation of this study was that the single-blind design may affect the interpretation of the results. Another limitation was that this study focused only on polyps between 5 and 15 mm in size, and polyps outside this range were not examined. Additionally, the retrieval of polyps requires the use of various disposable devices, including forceps/clips and snares, leading to a complex retrieval process and increased expenses. For single-polyp retrieval, the procedure typically lasts approximately 1–2 min. While it may not be time-efficient for retrieving a single polyp, we hypothesized that it could be time-efficient for patients with multiple polyps or those undergoing piecemeal EMR. The disposable devices were found to be expensive. Therefore, it is crucial to reduce costs and identify the most suitable indications for the use of polyp retrieval bags in future research. Furthermore, the use of different devices for polyp retrieval may impact fragmentation. However, the varying baseline characteristics and low fragmentation rates prevent the assessment of the potential influence of different devices on fragmentation. Additionally, this study cannot determine whether it is better to retrieve polyps after they are resected or to retrieve them while resecting for patients with multiple polyps. These issues should be explored in future research.

## Conclusions

In conclusion, in this single-center randomized controlled study, we demonstrated that polyp retrieval bags were effective for polyp retrieval and greatly decreased the fragmentation rate of polyps. Future studies should explore its ability to reduce the number of colonoscope insertions in patients with multiple and large (> 15 mm) polyps.

## Data Availability

No datasets were generated or analysed during the current study.
